# Communication Intervention Using Digital Technology to Facilitate Informed Choices at Childbirth in the Context of the COVID-19 Pandemic: Protocol for a Randomized Controlled Trial

**DOI:** 10.2196/25016

**Published:** 2021-05-21

**Authors:** Carmen Simone Grilo Diniz, Ana Carolina Arruda Franzon, Beatriz Fioretti-Foschi, Denise Yoshie Niy, Livia Sanches Pedrilio, Edson Amaro Jr, João Ricardo Sato

**Affiliations:** 1 School of Public Health University of Sao Paulo Sao Paulo Brazil; 2 Programa de Apoio ao Desenvolvimento Institucional do Sistema Único de Saúde Hospital Israelita Albert Einstein Sao Paulo Brazil; 3 Gender and Evidence on Maternity and Health Study Group School of Public Health University of Sao Paulo Sao Paulo Brazil; 4 Excellence Office Hospital Israelita Albert Einstein Sao Paulo Brazil; 5 Big Data Hospital Israelita Albert Einstein Sao Paulo Brazil; 6 Center of Mathematics, Computing and Cognition Universidade Federal do ABC Sao Bernardo do Campo Brazil

**Keywords:** app, childbirth, communication, COVID-19, health literacy, informatics, internet, intervention, maternal health, neonatal health, neonate public health, society, technology, women

## Abstract

**Background:**

In Brazil and other low- and middle-income countries, excess interventions in childbirth are associated with an increase in preterm and early-term births, contributing to stagnant morbidity and mortality of mothers and neonates. The fact that women often report a negative experience with vaginal childbirth, with physical pain and feelings of unsafety, neglect, or abuse, may explain the high acceptability of elective cesarean sections. The recognition of information needs and of the right to informed choice during childbirth can help change this reality. The internet has been the main source of health information, but its quality is highly variable.

**Objective:**

This study aimed to develop and evaluate an information and communication strategy through a smartphone app with respect to childbirth, to facilitate informed choices for access to safer and evidence-based care in the context of the COVID-19 pandemic.

**Methods:**

A randomized controlled trial, with 2 arms (intervention and control) and a closed, blind, parallel design, will be conducted with a smartphone app designed for behavior and opinion research in Brazil, with women of reproductive age previously registered on the app. After completing an entry questionnaire to verify the eligibility criteria and obtaining ethical consent, approximately 20,000 participants will be randomly allocated to the intervention and control groups at a 1:1 ratio. Participants allocated to the intervention group will be invited to engage in a digital information and communication strategy, which is designed to expand evidence-based knowledge on the advantages and disadvantages of options for labor and childbirth and the safety of the care processes. The information is based on the guidelines of the Ministry of Health and the World Health Organization for a positive childbirth experience and has been updated to include the new challenges and disruptions in maternity care within the context of the COVID-19 pandemic. The control group will receive information regarding disposable and reusable diapers as a placebo intervention. The groups will be compared in their responses in generating the birth plan and the entry and exit questionnaires, regarding responses less or more aligned with the guidelines for a positive childbirth experience. A qualitative component to map information needs is included.

**Results:**

The digital trial started recruiting participants in late October 2020, and data collection has been projected to be complete by December 2020.

**Conclusions:**

This study will evaluate an innovative intervention that has the potential to promote better communication between women and providers, such that they can make better choices using an approach suitable for use during the COVID-19 pandemic.

**Trial Registration:**

The Brazilian Clinical Trials Registry U1111-1255-8683; http://www.ensaiosclinicos.gov.br/rg/RBR-3g5f9f/

**International Registered Report Identifier (IRRID):**

PRR1-10.2196/25016

## Introduction

### Improvement of Maternal and Neonatal Health: a Permanent Challenge

Nationwide and worldwide studies have indicated, for many years, the need to act in order to reduce maternal and neonatal mortality, especially because a significant proportion of deaths can be avoided with adequate and timely assistance, considering the family planning continuum, prenatal care, childbirth assistance, and postnatal care [1-3]. Many sociohistorical, economic, and cultural factors interact, and the issue of maternal mortality remains poorly addressed, primarily in low- and middle-income countries such as Brazil. Thus, national and international stakeholders search for interventions that may contribute to significantly reduce maternal and neonatal mortality [1,3,4].

There have been advancements in the past decades in Brazil, with health professionals’ training, research, and improved access to prenatal care and childbirth assistance at health care facilities [5]. However, quality of care remains limited by non–evidence-based practices, within the context of increased use of inappropriate technology, which may be simultaneously harmful and costly [5]. At the health system level, in networks and facilities, women’s and families’ safety and well-being cannot be taken for granted, since recent studies have reported disrespectful practices in childbirth care [6,7], and in Brazil, infant and maternal mortalities have increased, raising public concern regarding maternity services [8]. In some scenarios, the uncertainties imposed by the COVID-19 pandemic in 2020 threaten the availability of medical resources and health care services, and studies have reported that Brazil has the highest COVID-19 mortality rates in the obstetric population [9,10].

### Maternal and Neonatal Morbidity and Mortality and Childbirth Care in Brazil

In Brazil, monitoring and regulation of obstetric interventions is limited. For example, the cesarean section rate remains as high as 55% since 2015, with rates of >80% in the private sector. This major surgery, when properly indicated and performed in a timely manner, protects the mother’s and the infant’s lives [11,12]. However, if performed without adequate reason, caesarean sections unnecessarily increase the risk to both mothers and neonates. In other words, unnecessary cesarian sections increase maternal and neonatal morbidity and mortality, as well as other negative outcomes, over the short and long term [13,14].

Several indicators show excess of interventions in childbirth care in Brazil, such as amniotomy (39.1%), fundal pressure (36.1%), episiotomy (53.5%), and oxytocin augmentation (36.4%) [5]. Simultaneously, pathological conditions are not adequately addressed, such as early identification of gestational hypertension, syphilis, and other infections during pregnancy. This is rather incompatible with current nationwide developments and with the presence of a universal health system.

Obstetric care can be characterized as a combination of “too little too late” with “too much too soon,” a reference to the insufficient and delayed use of necessary resources with the excessive and inopportune use of technology, potentially resulting in harm [15]. Abuse of unnecessary interventions has also been verified in the private sector, especially with high rates of caesarean sections. Furthermore, owing to the fact that in the private sector, a greater proportion of neonates are born in the early preterm period (37-39 weeks), neonatal admissions to intensive care units are more frequent, as are the cases of transient tachypnea, hypoglycemia, jaundice, and breastfeeding problems [5].

The main causes of neonatal death are prematurity (30.3%), congenital malformation (22.8%), and infection (18.5%), and mortality rates vary widely between regions. Late preterm infants (which accounts for 17.1% of all neonatal deaths) present a 9-fold greater chance of neonatal death compared to those born at term. In this context, it is recommended to improve the quality of prenatal care and to prevent iatrogenic prematurity, largely in cases of cesarean sections performed without technical indication [16]. Evidence- and rights-based care can reduce infant mortality by decreasing preventable neonatal deaths. This comprises deaths from intrapartum asphyxia, an important component of preventable neonatal deaths in the country [16].

### Resources to Increase Women’s Control Over Their Experience and Information Available on the Internet

During pregnancy, women seek information and want their needs to be heard, but educational materials, prenatal consultations, and support groups may not be sufficient or adequate [17]. Women can gather information from different sources such as their relatives, friends, the internet, and popular media [18-21]. Television shows and other popular media resources frequently resort to “experts” or other sources of information that are not scientifically validated. This influences women’s expectations and decisions regarding many aspects of their health, including childbirth fears and desires [19-23]. Incomplete information about cesarean sections is often disseminated, which “can lead women to underestimate important maternal and perinatal risks associated with this mode of parturition” [24]. A recent systematic review reported that there are few campaigns aimed at the general public to reduce the rates of cesarean sections [25].

The large volume of information available on the internet and its heterogeneity impact women's curiosity about certain topics, often to the detriment of others [26]. On Brazilian Portuguese webpages, the quality of information on cesarean sections varies from regular to poor, with low reliability and comprehensiveness [27]. This can cause anxiety among women [28], especially if the information is not discussed with health professionals [29] or confronted with other sources. To change this situation and contribute to increasing women's satisfaction, one possibility is to encourage the use of a birth plan, which improves their communication with health professionals and makes women more aware of their options [30].

In a systematic review in 2019, two trials observed that birth plans had a protective effect in promoting positive birth experiences in line with women’s perception. The quality of the studies was considered low, and the review concludes that more high-quality randomized studies are needed to assess the hypothesis that the use of the birth plan contributes to improving women’s satisfaction and promoting a more positive birth experience [31].

### Digital Technologies and Health Research

More than half of the world’s population has access to the Internet. In Brazil, a survey in June 2020 by the Getúlio Vargas Foundation of São Paulo [32] revealed that there are 190 million computers—including desktop, notebook, and tablet devices—currently in use in the country, which corresponds to 9 computers for every 10 inhabitants (90% per capita usage). Although approximately 25% of the population, especially the oldest and poorest individuals, remain digitally excluded, there are overall 234 million smartphones currently in use. In addition, considering notebook and tablet devices, there are 342 million portable devices currently in use; that is, 1.6 portable devices per inhabitant.

Throughout the 2010s, mobile phones spread digital health communication in low- and middle-income countries in South East Asia, sub-Saharan Africa, and Latin America, including Brazil. In the field of reproductive health, the establishment of opportunities for continuous communication, and complementary to institutional care for prenatal care, postpartum care and reproductive planning are associated with better perinatal outcomes [33,34], increased confidence and satisfaction with childbirth [35], increased knowledge of obstetric interventions such as uterine fundal pressure, episiotomy, and labor augmentation with oxytocin, increased perception of being better prepared for the childbirth experience [36], reduced anxiety [37], and reduced perinatal mortality [33]. Recent literature reviews revealed evidence of the effectiveness of digital interventions focused on pregnant and postpartum women, suggesting the need for more large-scale clinical trials [38,39].

To our knowledge, this will be a pioneering study, with population sampling in the country, assessing the preparation of a birth plan tool and women’s choices related to a safer and more positive childbirth experience.

### Emergence of the COVID-19 Pandemic: New Priorities Arise

On February 26, 2020, the first case of COVID-19 was reported in Brazil, and the disease was declared a pandemic by the World Health Organization on March 11, 2020. Since then, COVID-19 has monopolized the news and significantly modified the daily life of the population. Of note, Brazil ranks third worldwide with respect to the number of cases (>5 million) and deaths (>150,000) so far [40], while being amid a major political crisis. In addition, researchers in Brazil have observed an elevated COVID-19 mortality rate in the Brazilian obstetric population (12.7%), reporting 124 deaths among pregnant or postpartum women in June 2020, which is possibly the highest rate reported worldwide [9].

Innovative interventions have the potential to promote better communication between women in reproductive age and health care providers, such that they can make better choices. This study aims to develop and evaluate an information and communication strategy for a smartphone app on childbirth to facilitate informed choice for access to safer, more satisfactory, evidence-based assistance, in the context of the COVID-19 pandemic.

## Methods

### Study Design

This study is a randomized controlled trial, with 2 parallel arms (intervention and control) and a closed (only registered app users can participate) and blind design (only the participants are unaware of whether they are in the intervention or control arms). It is registered on the Brazilian Registry of Clinical Trials as “Communication intervention to improve informed choice at childbirth: a randomized controlled trial using digital technology in the context of the Covid-19 pandemic” with the World health Organization unique trial number U1111-1255-8683. The work is being conducted under a program supported by the Brazilian Ministry of Health (Programa de Apoio ao Desenvolvimento Institucional do Sistema Único de Saúde 25000.028646/2018-10).

### Study Setting

The study will be conducted through the restricted segment of a smartphone app. This app has been especially designed for behavior and opinion research in Brazil and will be used for participant recruitment, assessment of eligibility criteria, delivery and control of the intervention, and data collection.

Currently, the app has registered approximately 1,360,000 active users. Of them, 98,518 have accessed the internet in the past 14 days (September 2020); these individuals will be primarily invited to participate at the recruitment stage, reaching women with different socioeconomic and demographic characteristics in all States of Brazil.

All communication with participants will be carried out within the native and private environment of the app. Engagement will be voluntary, and participants will be financially reimbursed for the value of mobile internet use. The estimated compensation for participation in this study was set between R $5.00 and R $10.00 (US $0.92-$1.84), depending on the extent of participation, to cover the cost of mobile internet usage.

The stages of the study conducted through the app will be comprised of interactive questionnaires (called “missions” in the app), which are opportunities for engagement through direct communication with users. The questionnaires are as follows:

1. Eligibility criteria filter: the filter has 7 questions related to the study variables (intention to have biological children, number of children, those born through normal delivery, year of birth of the oldest child, race, education, and occupation) (Multimedia Appendix 1)
2. Invitation to participate in the study: information on the study and the investigators, objective of the study, and the dynamics of the missions (Multimedia Appendix 2)
3. Questionnaire to complete and validate the informed consent form: split across different screens, digitally validated by the participant (Multimedia Appendix 3)
4. Entry questionnaire: prospecting of values and preferences associated with the intended care (Multimedia Appendix 4)
5. Questionnaires on childbirth (intervention group) (Multimedia Appendix 5) or on diaper use (control group) (Multimedia Appendix 6)
6. Exit questionnaire: similar to the entry questionnaire (Multimedia Appendix 4)
7. Birth plan questionnaire (Multimedia Appendix 7)

### Participants

All women of reproductive age (18-49 years old) registered on the app will be notified of the invitation to participate in the study in order of priority.

### Eligibility Criteria

To be included in this study, a participant must be a registered user on the app; identify as a woman; be classified under class A, B, C, D, or E in accordance with the Brazil Criteria of 2015 of the Brazilian Association of Research Companies [41]; be aged between 18 and 49 years; and be pregnant or have biological children of any age or intend to have biological children in the future.

### Exclusion Criteria

Women without children and with no intention of having children in the future will be excluded from the study.

### Recruitment

A filter questionnaire will be administered to all women registered on the app and aged between 18 and 49 years, from all Brazilian States and socioeconomic strata. Those who are pregnant or have biological children of any age or intend to have biological children in the future will be notified of the invitation questionnaire to participate in the study. Thereafter, the informed consent questionnaire will be sent to all women who agree to participate in the study. The following stage comprises the entry questionnaire, and all women who digitally provide informed consent will be invited to answer it.

These questionnaires will account for sociodemographic data in addition to those already available on the app’s registry; type of health care assistance and funding (public or private, birth place including the hospital or health care institution, or professional including obstetricians and gynecologists, midwives, and nurse-midwives); clinical-obstetric characteristics (parity, mode of previous delivery, and the presence of a risk diagnosis); perception of safety in childbirth (for the mother and the neonate); and the perception of satisfaction or suffering expected during childbirth (for the mother and the neonate).

### Allocation and Randomization

Users will be recruited through the app, which is voluntarily installed by the user. Registrations are carried out organically, with no active screening for users and no advertising. Once registered, the new user answers questions related to his/her socioeconomic data. Missions will be sent to the profiles of users of all social classes (paired to represent the composition of the Brazilian population in the 5 regions of the country) and aged between 18 and 49 years.

Randomness in the sample is guaranteed in the program architecture of the missions, ensuring that those who undertake 1 mission will not be able to access the other mission, by blocking the mission ID through the execution of filters. On the app screen, the respondent sees only the name of the mission, with no indication of whether that participant is in the intervention or the control arm. To guarantee greater confidentiality and not induce interests or scams, we assigned the same name to the 2 missions: “Being a mother is making choices.” To balance the sample, the statistician team distributes the sample on the basis of the “n” defined for the study, thus generating a balance between the geographical region and economic class.

### Intervention

The intervention was developed on the basis of the guidelines for childbirth care from the Ministry of Health and the World Health Organization [42-44] updated with reference to the COVID-19 pandemic [45-47]. It is an educational resource to promote women’s knowledge of labor and childbirth care, as well as evidence-based choices for childbirth, which have been shown to protect maternal safety and satisfaction. The intervention consists of dummies, questions, and information cards developed and illustrated especially for this study by author BFF, a graphic artist specialized in maternal health.

**Figure 1:**
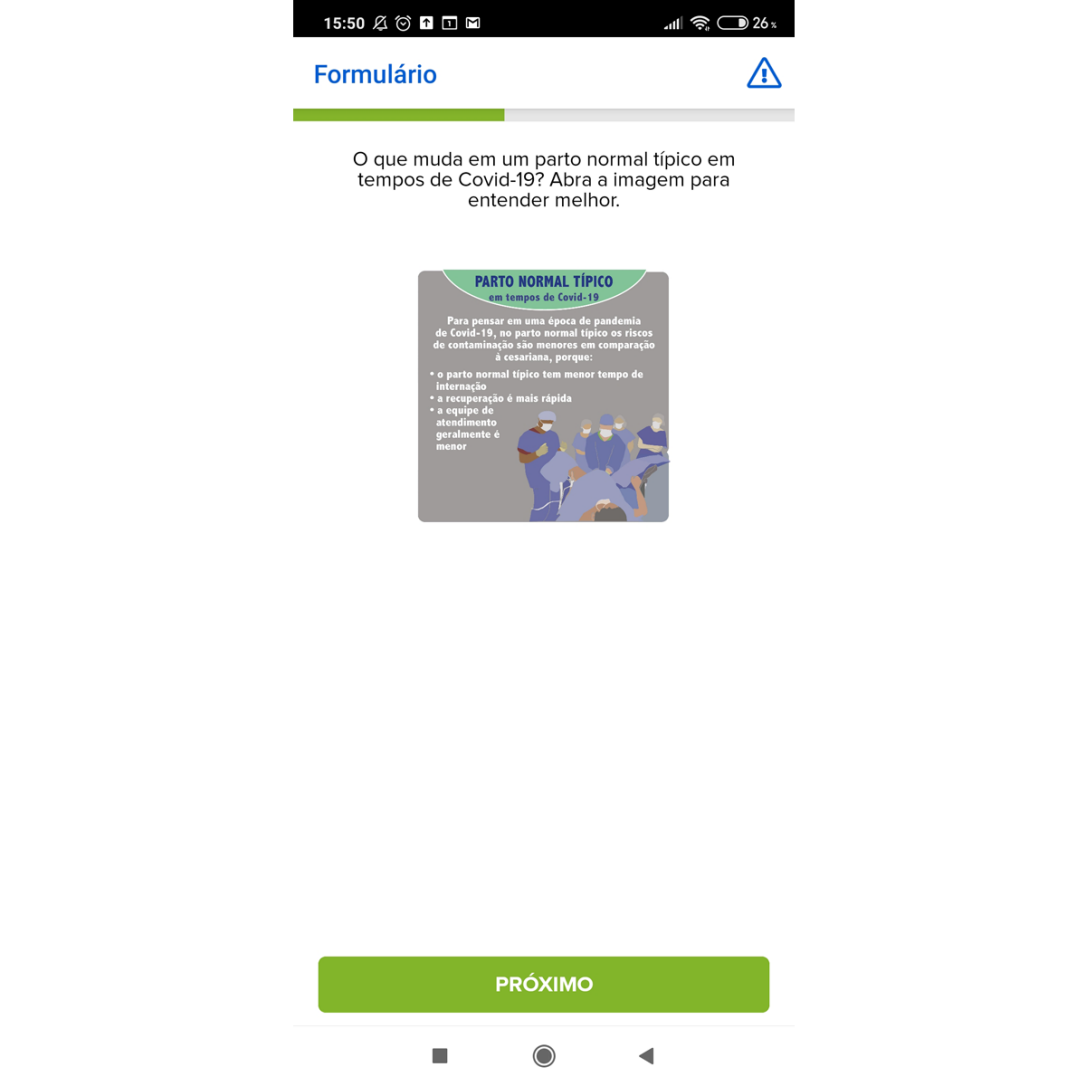
Screenshot of the app-based intervention: typical childbirth care and COVID-19.

The intervention presents information regarding the available models of care for childbirth, including information on care provider staff, obstetric practices in labor and childbirth, procedures for perinatal safety, skin-to-skin contact and breastfeeding in the first hour of life, and COVID-19 prevention procedures (Figure 1). The intervention requires the interaction and engagement of participants with the contents presented in a unique electronic “route” format, which will be available for engagement in a restricted segment of the app for 48 hours. After answering all questions, the women will be directed to 1 of the 6 cards with profiles associated with “childbirth styles” (Multimedia Appendix 8).

### Control or Comparator

Participants allocated to the control group will receive informative content on models of disposable and cloth diapers that are widely available in the Brazilian market. The theme of disposable diapers versus ecological diapers was selected for the control arm of this trial because it is related to choices in the maternity experience, without interfering with information on labor and birth provided in the intervention arm. The illustrated cards were developed on the basis of the following themes: advantages and disadvantages of each type of diaper; risks of allergies and dermatitis; chemical materials and components; ecological aspects used in manufacturing; decomposition, use of water; and practicality and costs (Multimedia Appendix 6).

### Birth Plan

The birth plan is a structured means of reflecting on the desired care for labor and birth, including an assessment of possibilities and uncertainties arising from the COVID-19 pandemic. It allows assessing the understanding of the intervention through questions on topics such as companionship, mobility, episiotomy, the Kristeller maneuver (fundal pressure), and augmentation with oxytocin. Upon completing the questionnaire (Multimedia Appendix 7), the participant sees her consolidated birth plan and is encouraged to take a screenshot to save this information.

Participants in both the intervention and control arms will be invited to elaborate a birth plan, and we will compare the differences in engagement and the responses.

### Adherence and Study Withdrawal

During recruitment, women of reproductive age will receive app notifications to take part in the study until sample size requirement is fulfilled. Throughout the study, the app will notify participants whenever they have to respond to a questionnaire, and they can save it to respond to it in the next 48 hours. This is intended to guarantee participant engagement in the study, as far it is comprised of 7 questionnaires.

Participants can opt out from the study at any time, withdrawing their consent to participate or simply by the digital act of “aborting a mission.”

### Outcomes

#### Primary Outcomes

The primary outcome measures (Table 1) will be the following:

1. Preference reported for the gestational outcome (for a healthy woman and neonate) the safety of which is determined on the basis of evidence: waiting for spontaneous onset of labor; waiting for the full 39 weeks; preference for vaginal childbirth; preference for cesarean delivery after labor; preference for elective cesarean delivery; or indifference.
2. Differences between the responses in the elaboration of the birth plan, aligned with the information provided in the intervention, in accordance with the Ministry of Health guidelines.
3. Knowledge of the COVID-19 pandemic’s impact in childbirth care and alternatives available.

**Table 1:** Outcomes of interest and definitions.

Endpoints	Definitions
Engagement in the intervention	Proportion of invited women who finish the missions
Reduced interest in elective cesarean section	Reduction in the proportion of women who intend to schedule an elective cesarean section
Increased engagement in seeking protagonism or informed choice	Proportion of women who develop a birth plan
Knowledge of the safest, most effective options associated with a more positive experience	Safer, more effective choices associated with a more positive experience in the exit survey and the birth plan
Engagement or desire to elaborate and share the experience and concerns	Elaboration of a narrative of the experience, doubts, and expectations regarding childbirth
Knowledge of the options available during the COVID-19 pandemic	Responses compatible with the information offered

### Participant Timeline

As the entire study will be carried out through a smartphone app, the study duration is shorter than that of regular trials. The first questionnaire (eligibility criteria filter) will be accessible to app users for 14 days. Once the user completes the questionnaire and is considered eligible for the study, her participation will last approximately 12 days (Multimedia Appendix 9).

### Parameters for Calculating the Sample Size

The sample size was calculated in accordance with the following parameters:

1. Study population size: in total, 32,000 women of reproductive age registered through the app (data collected from July 2019 before the onset of the COVID-19 pandemic).
2. Prevalence of the outcome of interest: a prevalence of 2.6% (Murcia, Spain) and 3% (São Paulo, Brazil) in the delivery rate of the birth plan to the obstetric care service.
3. Estimated impact of the intervention: a previous study that aimed to verify the effectiveness of a crowdsourcing strategy in promoting testing for hepatitis among men who have sex with men in China achieved a rate of 72.1% for the visualization of the multimedia components of the intervention. An increase in the number of tests performed was reported among 17.4% of the participants and confirmed by sending pictorial evidence of the examination among 7.9% of the participants [48]. In this study, a 20% impact on the outcome of preference for the elective cesarean section was estimated.
4. Margin of error and safety: considering that part of the parameters used during sampling was estimated from other studies and the possibility of loss to follow-up among a portion of the recruited women, a type I error (α) margin of 1% was adopted with a 99% confidence level, along with a safety margin for type II error (β) of 20%.

Thus, at least 9068 participants in each group will be required to demonstrate the effect of the educational intervention on the outcome measures of interest [49]. We expect to have 10,000 participants in each group. Expected recruiting numbers (approximate, depending on engagement) are detailed in Multimedia Appendix 9.

### Data Collection Methods

Collection of data on outcomes, baseline characteristics, birth plan, and other trial data will be collected automatically by the app. All study questionnaires were developed specifically for this study, and screenshots are available in Multimedia Appendices 1-8.

### Pilot Intervention and Adjustments

A pilot intervention was conducted with 1000 participants prior to the final study for final adjustment of the questionnaires’ linguistic adequacy and to evaluate the optimal sequence of missions to be administered to the participants. Engagement in the study questionnaires was higher than expected.

### Data Management

For this study, a series of data management procedures will be implemented to ensure data protection, safety, privacy, and confidentiality. Data management will target the following three aspects: participant data treatment by the app’s parent company, relevant information transfer to researchers, and data set maintenance by all teams. It is worth mentioning that in compliance with security measures established by the general data protection law [50,51] and the resolutions of the National Council of Health for human experimentation [52], researchers will receive only the variables of interest for the study.

At the individual level, the collected data will be computed directly in the parent company’s system. This will include the following: verification of the individual's registration, deidentification of the data and separation of the population data for this study from the rest of the platform data, and the creation of an individual cloud to store the data for this specific study. Only the variables of interest in the deidentified data of the study participants will be used.

When accepting the app’s terms of use and privacy policy, all users have their data automatically pseudo-anonymized (ie, unidentifiable). These data include the following: personal data collected upon registration; complementary data captured during app use, such as a version of the smartphone’s operating system or device model; and all user responses during interventions.

At the level of database management by the researchers, the file will be initially processed within the app’s servers, where the raw data files will also be stored and will not be used for any purpose other than this study.

### Data Analysis Plan

#### Statistical Analysis

CONSORT (consolidated standards of reporting trials) guidelines will be used in reporting the results. An intention-to-treat analysis will be performed to compare data from the study's entry and exit surveys. This approach promotes a pragmatic assessment of the potential benefits of the intervention, as it incorporates loss to follow-up in the analyzed data of the intervention group.

Sociodemographic characteristics will be analyzed using descriptive statistics; between-group comparisons will be performed using chi-square analysis or a 2-tailed *t* test. For all group comparisons, the results shall be expressed as an effect (or relative risk for binary outcomes), corresponding 2-sided 99% confidence intervals (α=1%; power=80%), and associated *P* values. Adjusted analyses using baseline variables shall be performed using regression analysis to determine the continuing influence of key baseline characteristics on the outcomes, including female app users in the public and private sectors, cesarean sections, and vaginal delivery. The analyses will be conducted using the R statistical analysis software (The R Foundation) [53].

#### Qualitative Analysis

Audio narratives (entry and exit questionnaires and birth plan) will be automatically transcribed and analyzed in accordance with Bardin thematic analysis [54], using the Qualitative Solutions Research NVivo software (version 12.0, QSR International). A priori categories will include the following: feeling informed to make choices, feeling physically and emotionally secure, factors influencing satisfaction or dissatisfaction, desire to do something differently, and information needs. A posteriori categories are expected to emerge, as experience is considered an expansive learning opportunity [55].

### Data Monitoring and Auditing

Since this is an educational intervention with low risk among participants, no data monitoring committee has been contemplated. There will be no independent review of trial processes (data auditing) during its execution, as the whole trial will last less than a month and routine data quality management will be performed.

### Potential Risks to Participants

In this type of study, minimum risks are expected for the participants, such as the following: fatigue during the missions due to the long duration of the study or the lack of motivation with the topic of interest in the questionnaires; and discomfort or embarrassment if the participant is unaware of any term or situation presented during the study, despite efforts to adapt the language.

Another risk is the loss of data confidentiality, which will be minimized with protection measures by the researchers, as outlined during data collection and management. At the app level, users have preserved and guaranteed the rights to their data under the terms of the Brazilian General Data Protection Law.

## Results

The digital trial started recruiting participants in late October 2020, and data collection has been projected to be completed by December 2020.

The study will be conducted in accordance with Resolutions 466/2012 and 510/2016 of the National Health Council [52,56]. Information regarding personal interest will be obtained exclusively for the study, and privacy, confidentiality, and preservation of the participants’ identity will be ensured. Furthermore, the protection of data of all users of the digital platform, where the intervention will be applied, comply with the procedures outlined in accordance with the Brazilian General Data Protection Law.

Participation in the study must be voluntary and will be confirmed only after digital validation of the informed consent form. The study was approved by the institutional review board of Hospital Israelita Albert Einstein (project# 4194-20) and by the National Commission for Ethics in Research of the National Health Council of the Ministry of Health (CAAE: 32840920.7.0000.0071).

App users will individually provide consent to participate in the study. The risk to confidentiality will be minimized through protection measures by the researchers. All participants’ personal data and responses to participation will be kept private and confidential, thus guaranteeing anonymity, and at no time will participant identity be disclosed.

The data will be accessed only by the investigators. Since this is an educational intervention, it is of low risk, and no adverse effects are expected, and no compensation for study participation is contemplated.

## Discussion

There are presently no intentions to implement major protocol modifications; in case they occur, relevant parties will be solicited.

No restrictions are anticipated among investigators and sponsors to communicate trial results. We expect to disseminate the outcomes to the general public, health providers, and through scientific publications by means of a joint collaboration of the School of Public Health, University of São Paulo, with Sociedade Beneficente Israelita Brasileira Hospital Albert Einstein. The research teams from both institutions will author the resulting publications, with no intention to use professional manuscript writing services.

We will request authorization from the sponsor to grant public access to the complete protocol, anonymized participant-level data set, and the statistical code, 2 years after the completion of the study for both research and educational purposes, and to explore new hypotheses arising from the study.

If successful, this educative intervention (which can be used outside of the smartphone app) will be made publicly accessible after the dissemination of results, with proper credit and recognition.

## Appendix

Multimedia Appendix 1 Eligibility criteria filter questionnaire screenshot.

Multimedia Appendix 2 Invitation questionnaire screenshot.

Multimedia Appendix 3 Informed consent questionnaire screenshot.

Multimedia Appendix 4 Entry and exit questionnaire screenshot.

Multimedia Appendix 5 Intervention questionnaire screenshot.

Multimedia Appendix 6 Control questionnaire screenshot.

Multimedia Appendix 7 Birth plan questionnaire screenshot.

Multimedia Appendix 8 Childbirth styles screenshot.

Multimedia Appendix 9 Schedule of enrolment, interventions, and assessments.
