# Supplementary figures and images for: Communication Intervention Using Digital Technology to Facilitate Informed Choices at Childbirth in the Context of the COVID-19 Pandemic: Protocol for a Randomized Controlled Trial
Source: JMIR Res Protoc. 2021 May 21;10(5):e25016. doi: 10.2196/25016 (PMC8143871; doi:10.2196/25016)

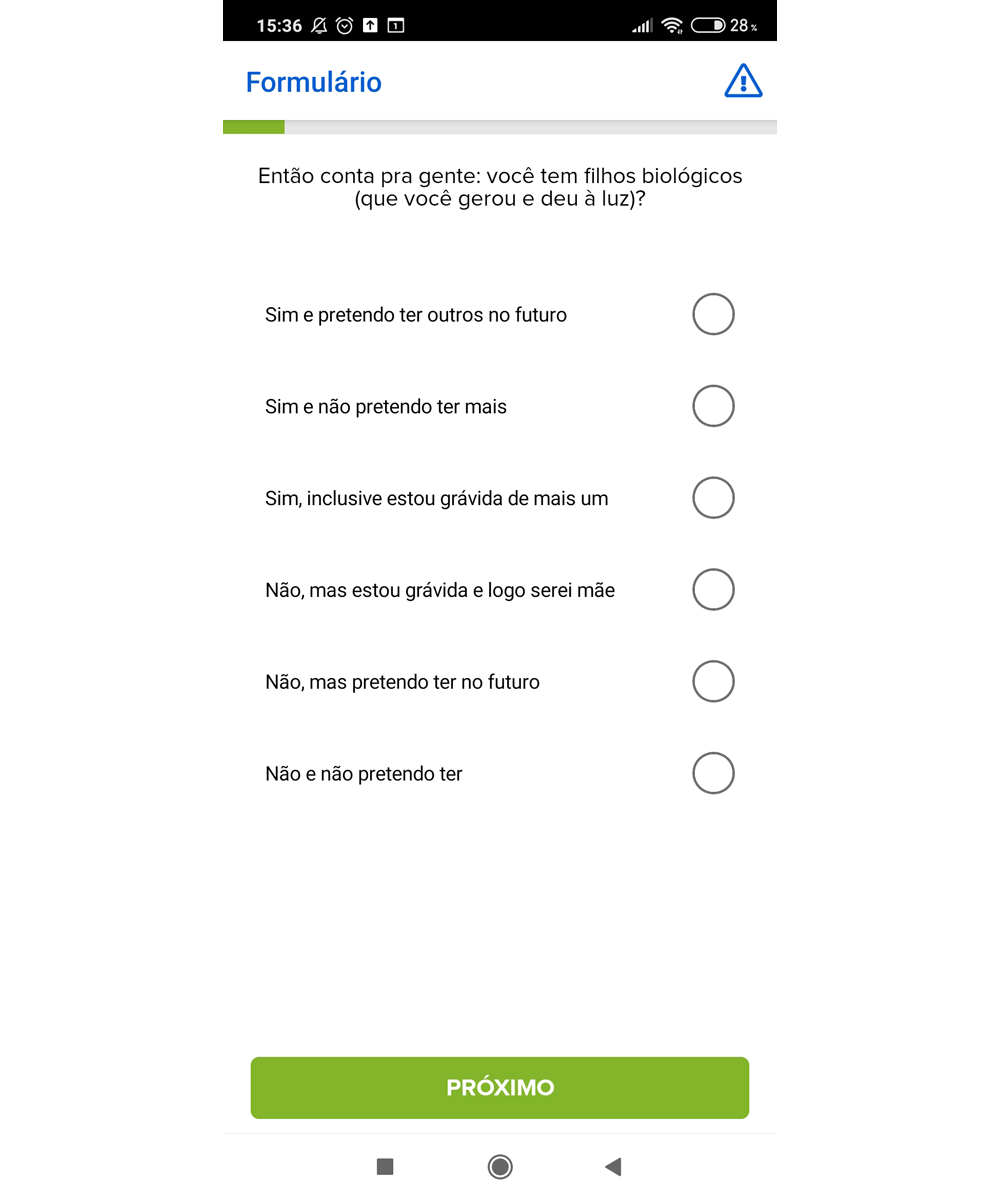

Supplement: Multimedia Appendix 1 [file resprot_v10i5e25016_app1.png]

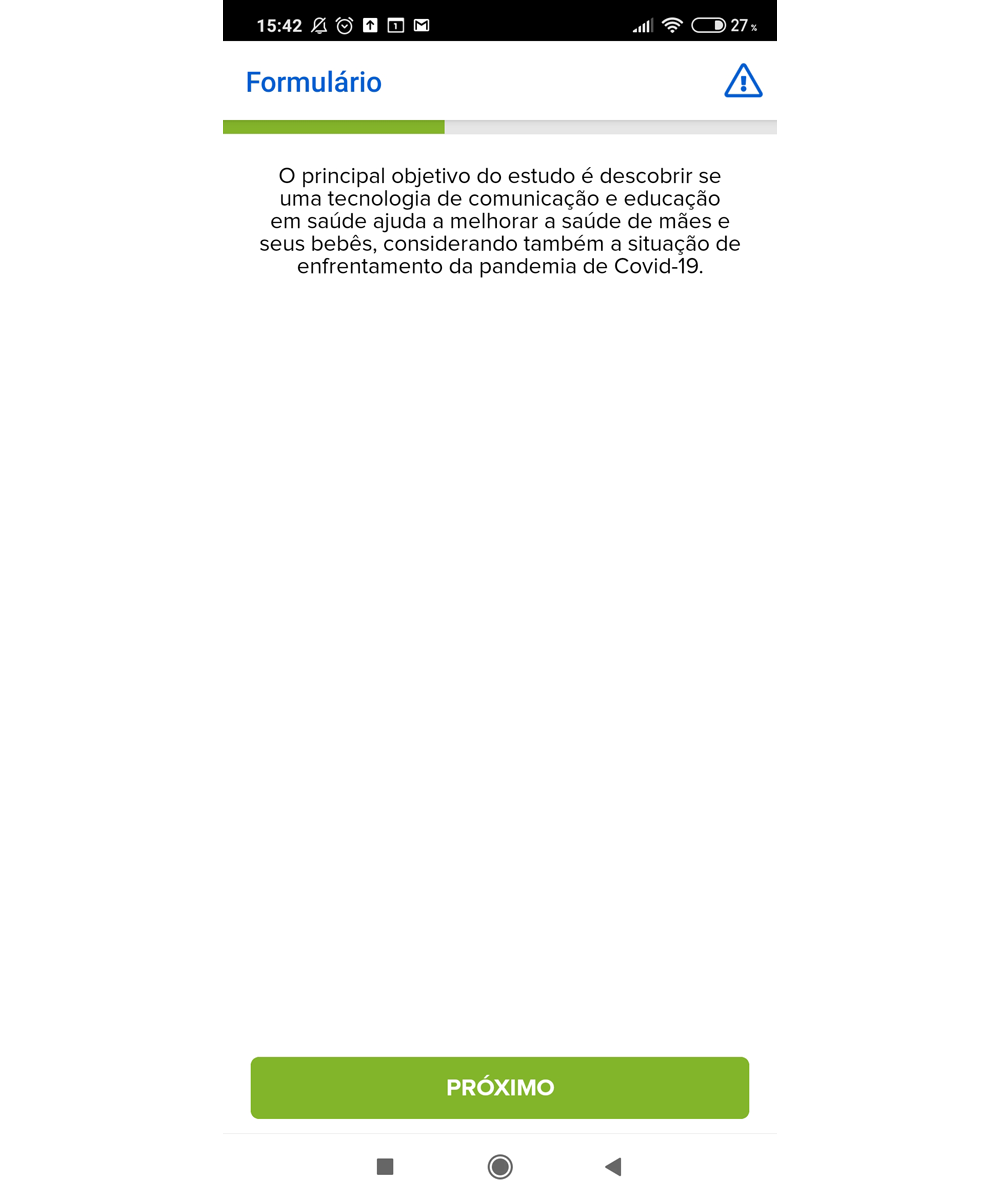

Supplement: Multimedia Appendix 2 [file resprot_v10i5e25016_app2.png]

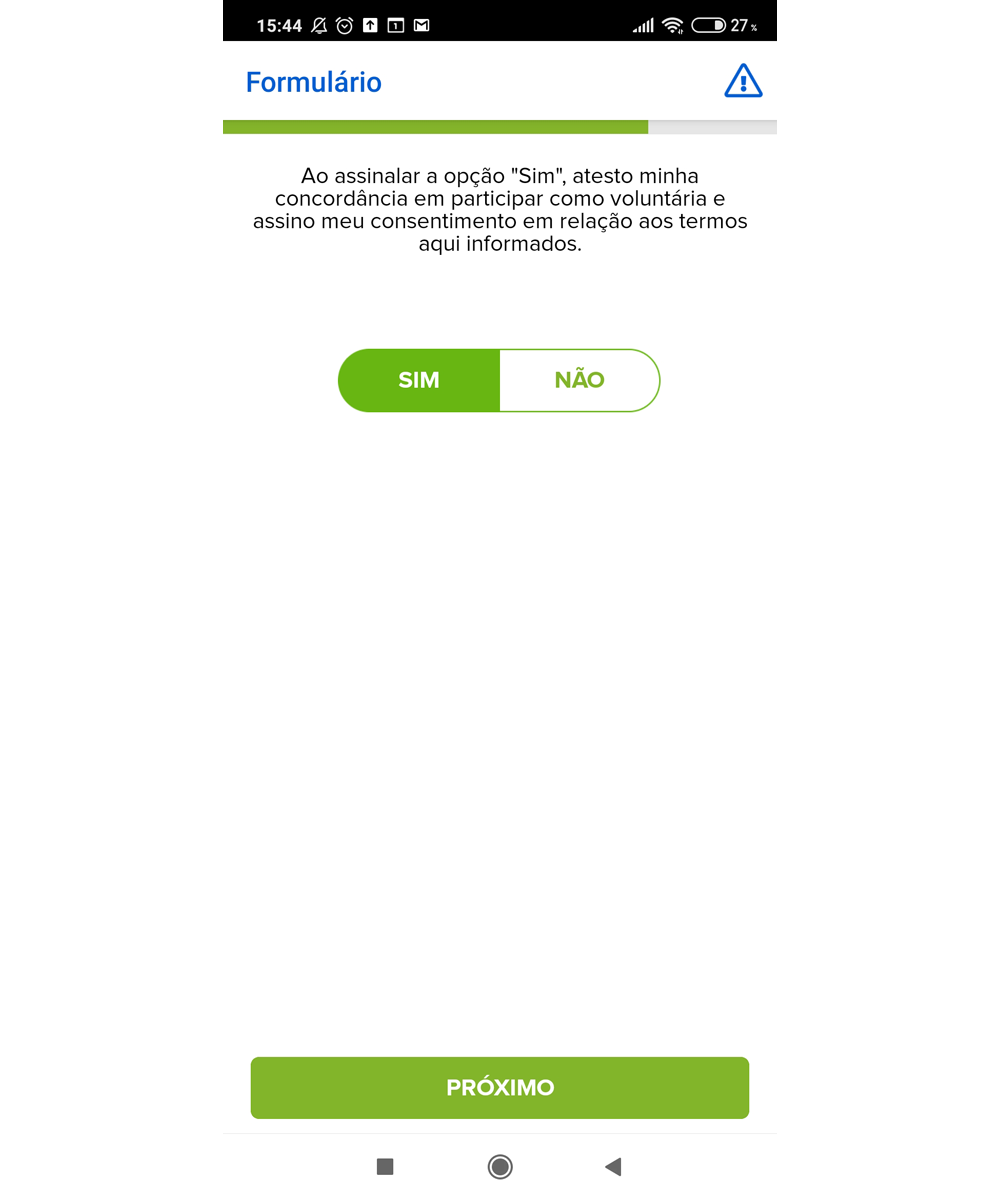

Supplement: Multimedia Appendix 3 [file resprot_v10i5e25016_app3.png]

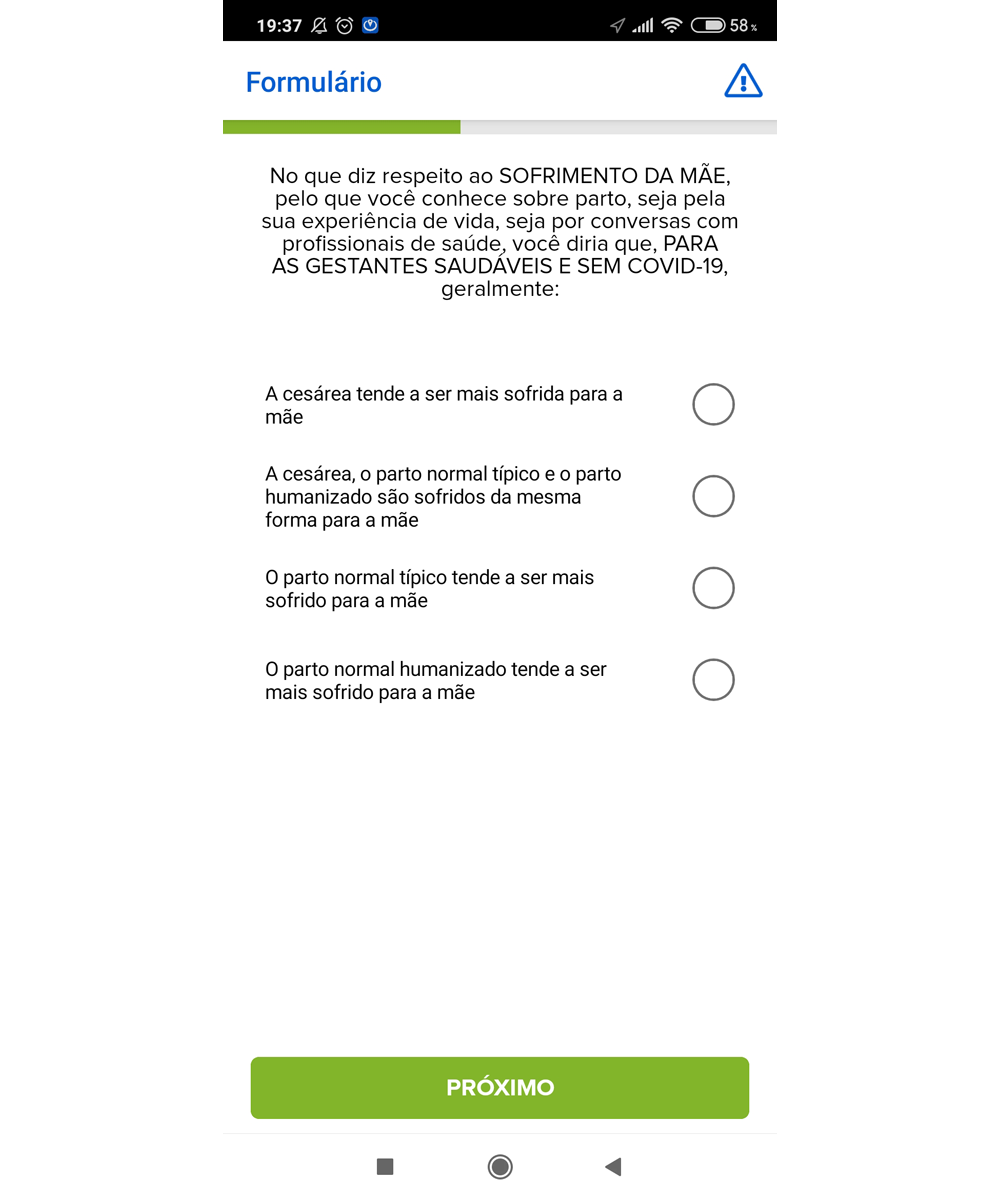

Supplement: Multimedia Appendix 4 [file resprot_v10i5e25016_app4.png]

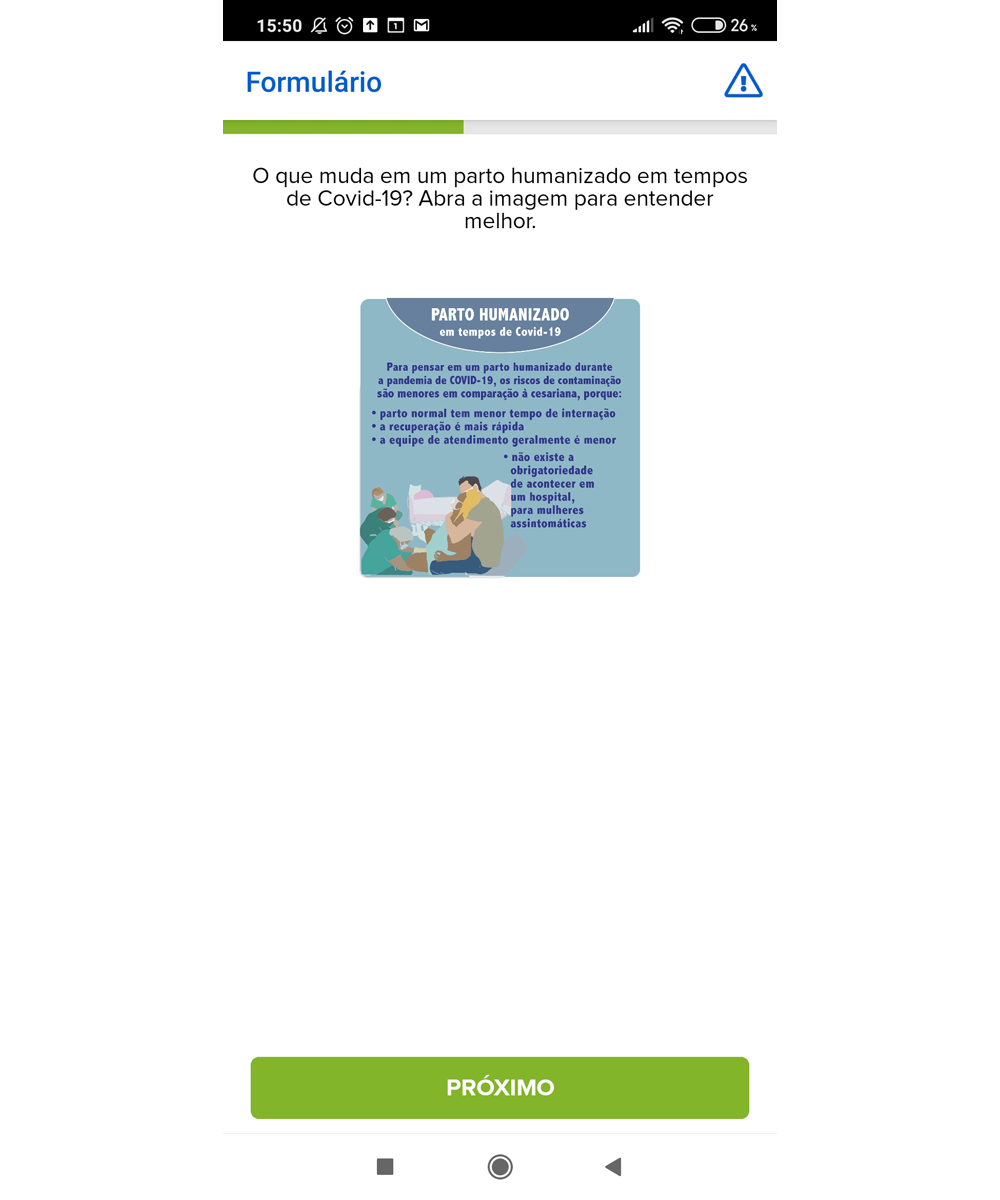

Supplement: Multimedia Appendix 5 [file resprot_v10i5e25016_app5.png]

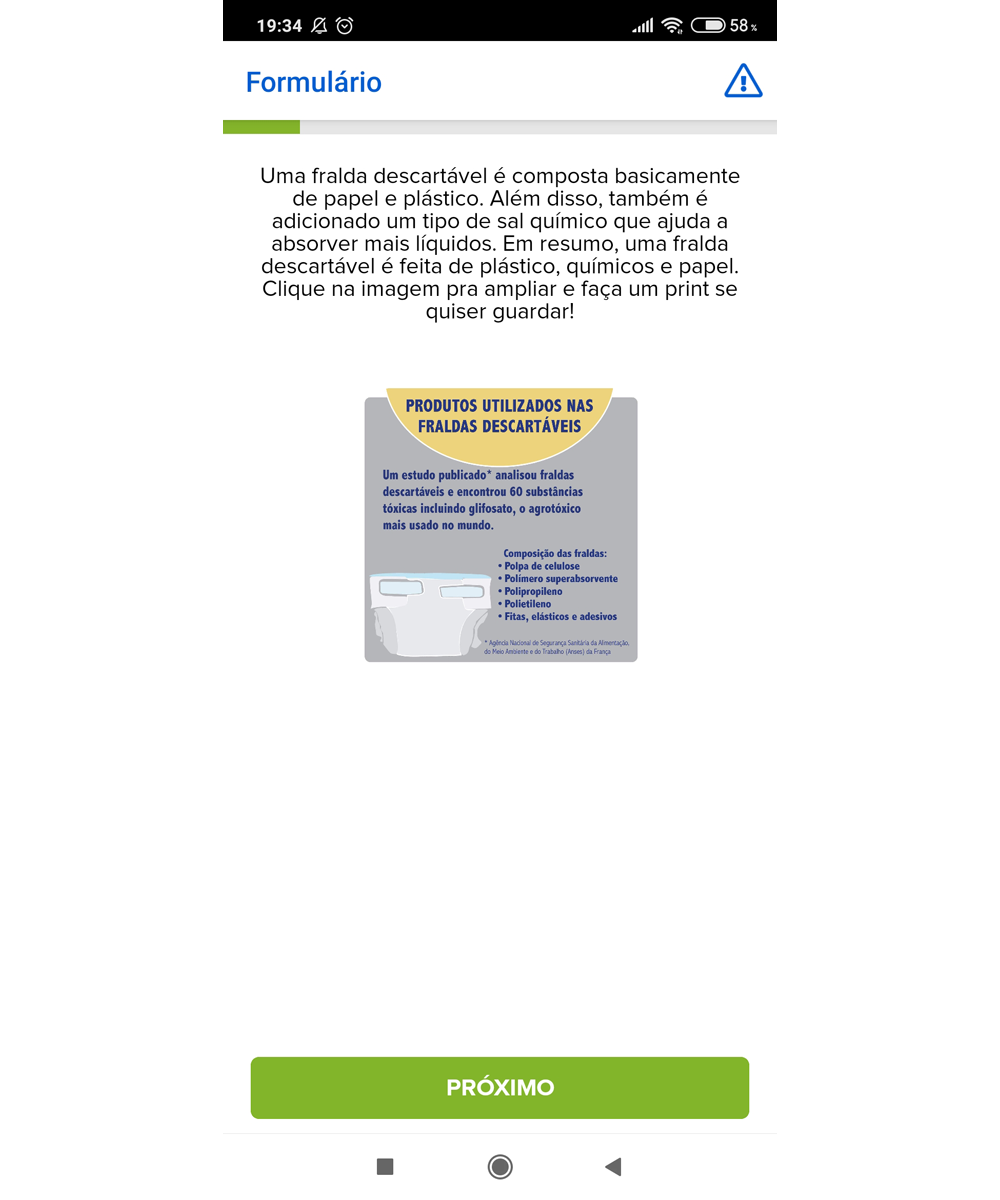

Supplement: Multimedia Appendix 6 [file resprot_v10i5e25016_app6.png]

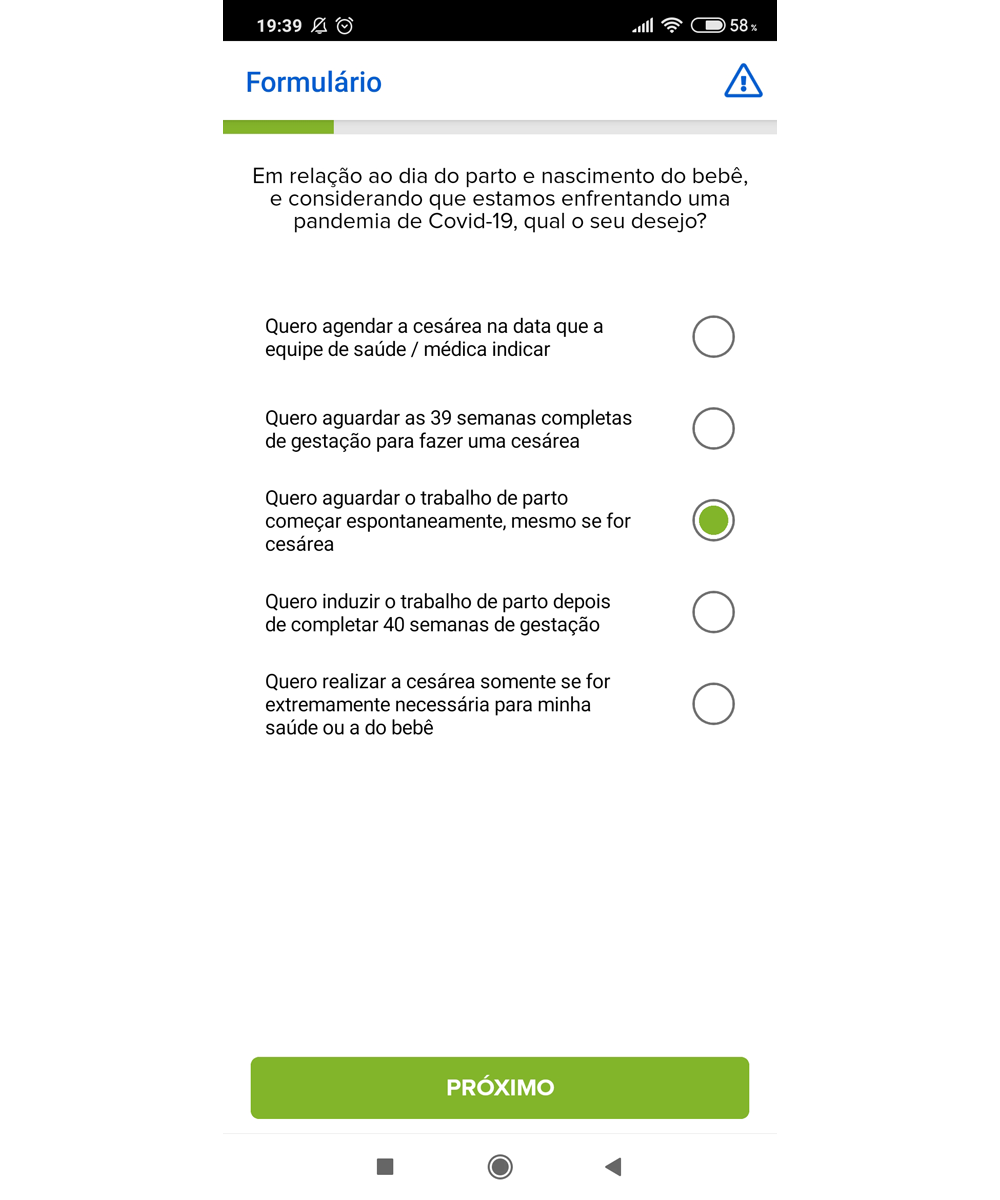

Supplement: Multimedia Appendix 7 [file resprot_v10i5e25016_app7.png]

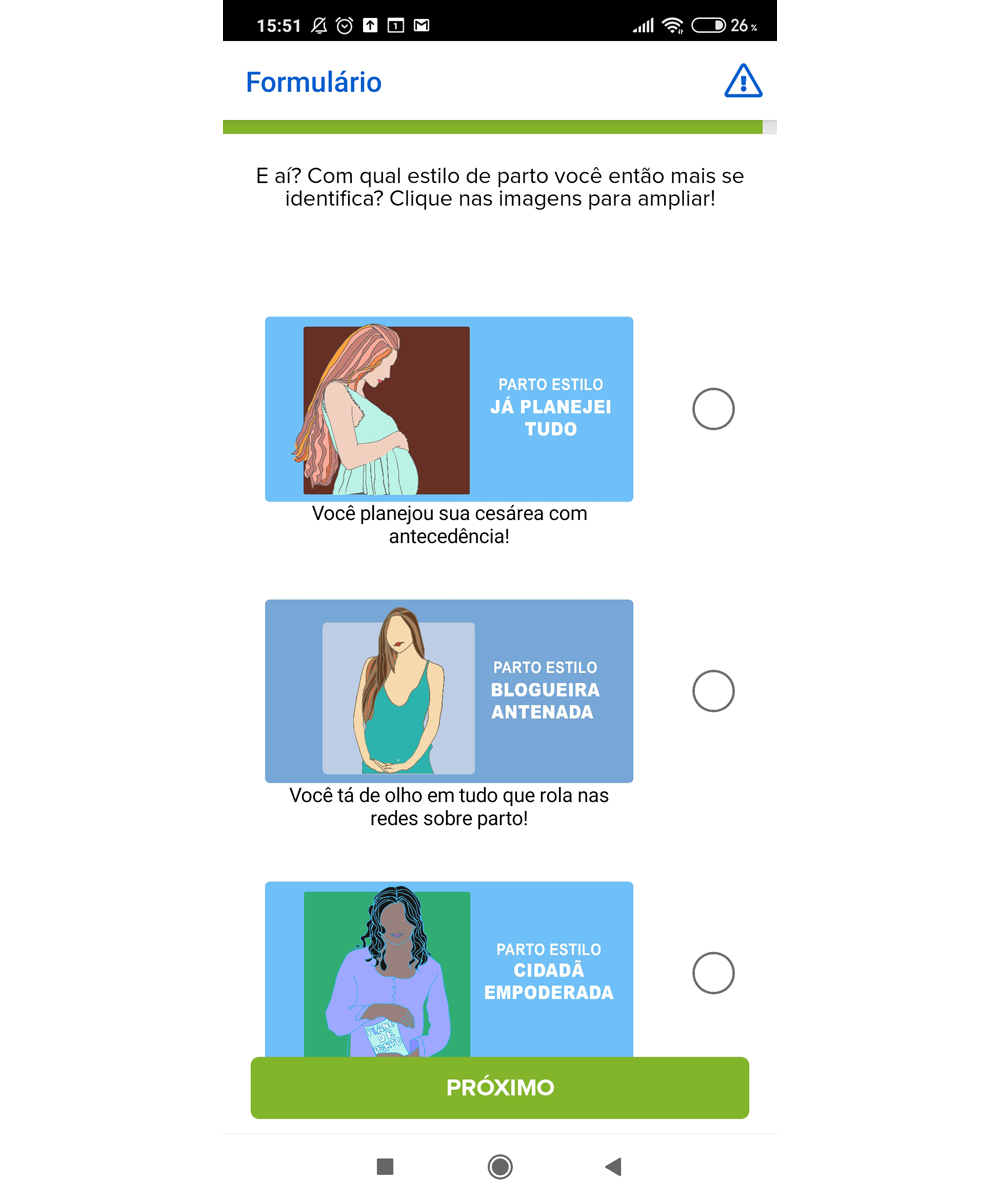

Supplement: Multimedia Appendix 8 [file resprot_v10i5e25016_app8.png]
