# Supplementary material for: Communication Intervention Using Digital Technology to Facilitate Informed Choices at Childbirth in the Context of the COVID-19 Pandemic: Protocol for a Randomized Controlled Trial
Source: JMIR Res Protoc. 2021 May 21;10(5):e25016. doi: 10.2196/25016 (PMC8143871; doi:10.2196/25016)
Supplement: Multimedia Appendix 9 [file resprot_v10i5e25016_app9.pdf]

MULTIMEDIA APPENDIX 9: Schedule of enrolment, interventions, and assessments

	**STUDY PERIOD**							
	**Enrollment**	**Allocation**	**Post-allocation**					**Close-out**
TIMEPOINT (days)	***-D-4***	**0**	***D2***	***D4***	***D6***	***D8***	***D10***	***D12***
**ENROLLMENT:**								
Recruitment (n=32,000)	X							
Eligibility screen (n=26,000)	X							
Invitation to enroll (24,000)	X							
Informed consent (20,000)	X							
Random allocation		X						
**INTERVENTIONS:**								
Entry questionnaire (n=20,000)			X					
Childbirth health communication intervention (n=10,000)				◆——	——	——◆		
Diapers placebo intervention (n=10,000)				◆——	——	——◆		
Exit questionnaire (n=18,000)							X	
Birth plan questionnaire (n=18,000)								X
**ASSESSMENTS:**								
Sociodemographics and obstetrics	X							
Engagement in the intervention	X		X	X	X	X	X	X
Reduced interest in elective cesarean section							X	X
Increased engagement in seeking protagonism / informed choice			X	X	X	X	X	X
Knowledge of the safest, most effective options associated with a more positive experience							X	X
Engagement / Desire to elaborate and share the experience and concerns						X		X
Knowledge of the options available in the Covid-19 pandemic						X	X	X
Qualitative data opportunity			X				X	X
